# Phase I studies assessing safety and pharmacokinetics of nacubactam administered alone or in combination with cefepime or aztreonam in Japanese healthy participants

**DOI:** 10.1128/aac.01770-25

**Published:** 2026-04-13

**Authors:** Jun Morita, Noriko Hida, Takuma Yonemura, Taigi Yamazaki, Hiroki Sato, Masayo Sumiya, Risako Takaya, Yuji Kumagai, Naoki Uchida

**Affiliations:** 1 R & D Division, Meiji Seika Pharma Co., Ltd. 13418 , Chuo-ku, Tokyo, Japan; 2 Department of Clinical Research and Development, Graduate School of Pharmacy, Showa Medical University 13059 , Setagaya-ku, Tokyo, Japan; 3 SHOWA Medical University Clinical Research Institute for Clinical Pharmacology and Therapeutics 13059 , Setagaya-ku, Tokyo, Japan; 4 Souseikai Sumida Hospital, Tokyo, Japan; 5 Kitasato University Kitasato Institute Hospital 12877, Minato-ku, Tokyo, Japan; 6 Department of Clinical Pharmacology, Graduate School of Medicine, SHOWA Medical University 13059 , Setagaya-ku, Tokyo, Japan; University of Houston, Houston, Texas, USA

**Keywords:** nacubactam, beta-lactamase inhibitor, clinical trial, phase 1, pharmacokinetics, safety, cefepime, aztreonam, Japanese

## Abstract

Nacubactam is a novel developed β-lactamase inhibitor. Two randomized, double-blind, placebo-controlled phase 1 studies (OP0595-2 and -4 studies) were conducted to evaluate its pharmacokinetics and safety in healthy Japanese male participants. In the OP0595-2 study, single ascending doses (1, 2, and 4 g) and multiple doses (1 and 2 g per dose for 7 days) of nacubactam were administered intravenously over 90 min. In the OP0595-4 study, 2 g of nacubactam was administered intravenously over 60 min for 7 days in combination with cefepime or aztreonam (2 g per dose each). In both studies, participants were randomized in a 3:1 ratio to receive nacubactam or placebo. In the OP0595-2 study, exposure to nacubactam increased in a dose-dependent manner following single infusion, with steady mean trough plasma concentrations observed after Day 4 during multiple dosing. Metabolites of nacubactam were detected at low levels compared to the parent drug. Nacubactam was predominantly excreted unchanged in the urine, indicating minimal metabolic clearance. In the OP0595-4 study, administration of cefepime or aztreonam did not alter the pharmacokinetic profile of nacubactam. In both studies, nacubactam, whether administered alone or combined with cefepime or aztreonam, was generally well tolerated. These favorable findings support further clinical development of nacubactam.

## INTRODUCTION

Antimicrobial-resistant bacteria pose a global threat to morbidity and mortality (1). Among them, multidrug-resistant Gram-negative bacteria are a major health concern (2, 3). β-Lactam antibiotics are the cornerstone for bacterial treatment; however, resistance in Gram-negative bacteria is mainly due to β-lactamases (4). Thus, monotherapy with carbapenem—a class of β-lactam antibiotics with potent activity—may no longer be an appropriate treatment option (5). Under these circumstances, the development of an effective β-lactamase inhibitor has been needed.

Nacubactam (also known as OP0595) is a diazabicyclooctane developed by Meiji Seika Pharma Co., Ltd. (Tokyo, Japan). It strongly inhibits Class A and C β-lactamase (6). In addition, it binds penicillin binding protein 2 of *Enterobacteriaceae* and enhances the antimicrobial activity of various β-lactam antibiotics (6). Therefore, combining nacubactam with β-lactam antibiotics may enhance antimicrobial activity. In fact, the previous non-clinical studies showed that the combination of these agents was active against bacteria producing any class of β-lactamase (7–11). These favorable profiles of nacubactam have led to the clinical development.

**Fig 1:**
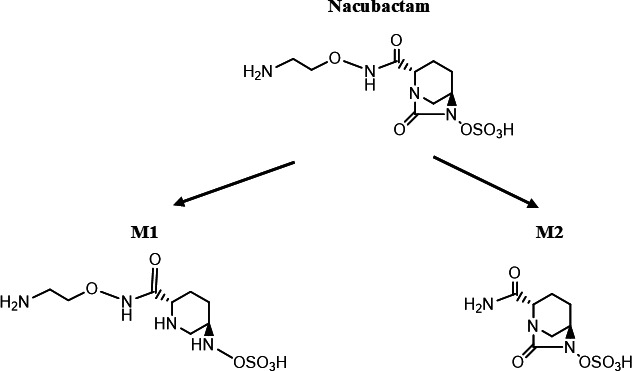
Nacubactam metabolic pathways in human.

Recently, nacubactam’s pharmacokinetics and safety were assessed in single and multiple ascending dose studies in healthy participants (12). In these studies, nacubactam was generally well tolerated and its pharmacokinetics appeared linear across the dose range investigated. The multiple ascending dose study, in which nacubactam was administered alone or in combination with meropenem, also showed the absence of drug-drug interactions between nacubactam and meropenem, a broad-spectrum carbapenem used to treat a variety of bacterial infections. In plasma and urine, metabolites were found after IV administration of nacubactam. Nacubactam was metabolized to M1 and M2 (Fig. 1). The metabolites observed were M1 (open-ring form) which has no inhibitory activity and M2 (deaminoethoxy form) has inhibitory activity (Class A, C, and D β-lactamases) (6, 12).

To support the conduct of a Phase 3 study to confirm efficacy and safety, including Japanese patients, we conducted these studies to demonstrate that pharmacokinetics and safety in the Japanese population are comparable to those observed in non-Japanese patients. These studies were conducted under the assumption that there would be no racial differences due to renally excreted nacubactam (12). Based on these findings, we conducted two randomized, double-blind, placebo-controlled phase 1 studies (OP0595-2 and -4 studies) in Japan. The objective of the OP0595-2 study was to assess the safety, tolerability, and pharmacokinetic (PK) profiles of nacubactam following the single and multiple doses. The objective of the OP0595-4 study was to assess the safety, tolerability, and PK profiles following the multiple doses of nacubactam coadministered with cefepime or aztreonam. Cefepime is stable against Class D β-lactamase (13), while aztreonam is stable against Class B enzyme (14). Nacubactam has no inhibitory activity against class B β-lactamase (MBL), but Aztreonam is stable against class B β-lactamase, Aztreonam/nacubactam exhibits strong antibacterial activity against MBL-producing bacteria. Nacubactam has some inhibitory activity against class D β-lactamase, but this inhibitory activity is weaker than that of classes A and C. Because cefepime is stable against class D β-lactamase (MBL), cefepime/nacubactam exhibits strong antibacterial activity against class D β-lactamase-producing bacteria. Thus, these agents were considered to complement the antimicrobial activity of nacubactam.

## RESULTS

### Participant disposition and baseline characteristics

**Fig 2:**
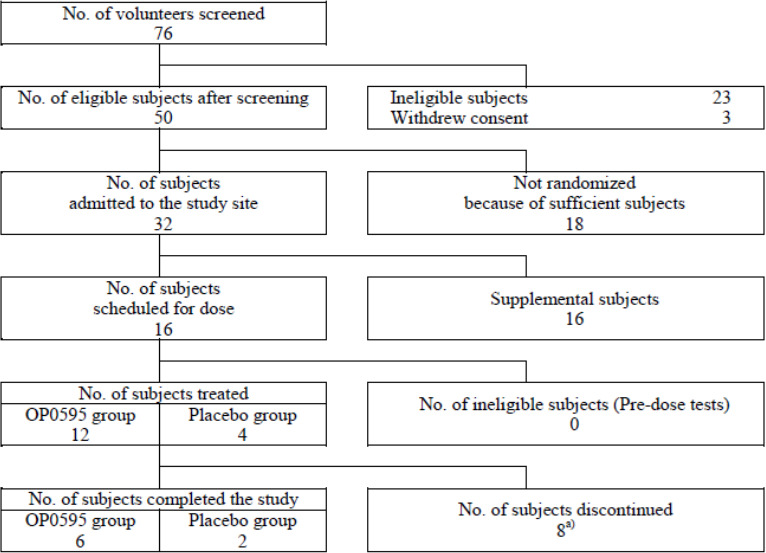
Disposition of subjects in step 1 (OP0595-2 study).

In Step 1 of the OP0595-2 study, eight participants (six nacubactam, two placebo) were planned for enrollment. However, four participants (three nacubactam, one placebo) discontinued on Day 2 due to incorrect dosing on Day 1. Another four participants (three nacubactam, one placebo) discontinued prior to the third infusion on Day 7 (5th multiple-dose day) due to foreign substance found in the drug vial. The observed substance was produced by coring when the injection needle punctured the rubber stopper of the vial. These eight participants were replaced (Fig. 2). Steps 2 and 3 each enrolled eight participants (six nacubactam, two placebo), all of whom completed the study.

Participants discontinuing on Day 2 in Step 1 were excluded from safety and PK analyses, with no reported treatment emergent adverse events (TEAEs). Those discontinuing on Day 7 were included in the safety analysis; among them, three participants who received nacubactam until Day 7 were included in single-dose PK analysis but excluded from multiple-dose PK analysis. Ultimately, the safety analysis included 28 participants (9 in the 1 g, 6 in 2 g, 6 in 4 g, and 7 placebo groups). Of these, 21 participants who received nacubactam were included in single-dose PK analysis; 12 completers (6 in each 1 and 2 g groups) were included in multiple-dose PK analysis.

Each OP0595-4 cohort enrolled eight participants (six nacubactam with concomitant drug, two placebo). One participant in Cohort 1 discontinued due to TEAE (seborrheic dermatitis) on Day 2. All Cohort 2 participants discontinued post Day 8 examination due to Japan’s COVID-19 State of Emergency declaration on 26 February 2021 (https://japan.kantei.go.jp/ongoingtopics/_00043.html). All participants (six in the nacubactam with cefepime group, six in the nacubactam with aztreonam group, and four in the placebo group) were included in the safety analysis. Of these, 12 participants who received nacubactam in combination with cefepime or aztreonam were included in the PK analysis.

Baseline characteristics were balanced across treatment groups in both studies (Tables 1 and 2, respectively). Mean ages ranged from 26.0 to 29.0 years in the OP0595-2 study and from 24.5 to 26.0 years in the OP0595-4 study; mean weight ranged from 60.70 to 64.48 kg and from 61.98 to 67.92 kg, respectively.

**TABLE 1:** Baseline characteristics of the study participants in the OP0595-2 study^*a*^

Item	Descriptive statistics	Total	Nacubactam 1 g	Nacubactam 2 g	Nacubactam 4 g	Placebo
		(*n* = 28)	(*n* = 9)	(*n* = 6)	(*n* = 6)	(*n* = 7)
Age (year)	Mean	27.0	26.0	26.0	29.0	27.6
	SD	6.1	5.2	6.8	7.2	6.8
Height (cm)	Mean	170.59	172.86	167.38	170.80	170.26
	SD	4.98	5.45	3.98	4.00	5.24
Weight (kg)	Mean	62.79	64.48	62.98	60.70	62.26
	SD	5.97	5.49	2.82	4.53	9.34
BMI (kg/m^2^)	Mean	21.57	21.60	22.48	20.82	21.39
	SD	1.68	1.75	0.85	1.47	2.19

^*a*^ SD, standard deviation; BMI, body mass index.

**TABLE 2:** Baseline characteristics of the study participants in the OP0595-4 study^*a*^

Item	Descriptive statistics	Nacubactam 2 g with Cefepime 2 g	Nacubactam 2 g with Aztreonam 2 g	Placebo
		(*n* = 6)	(*n* = 6)	(*n* = 4)
Age	Mean	25.7	24.5	26.0
(year)	SD	7.1	3.0	8.8
Height	Mean	172.75	173.50	171.13
(cm)	SD	4.50	4.86	4.88
Weight	Mean	67.92	67.88	61.98
(kg)	SD	5.93	7.91	5.54
BMI	Mean	22.67	22.45	21.10
(kg/m^2^)	SD	1.27	1.76	1.00

^*a*^ SD, standard deviation; BMI, body mass index.

### Pharmacokinetics

#### Nacubactam in plasma concentrations

**Fig 3:**
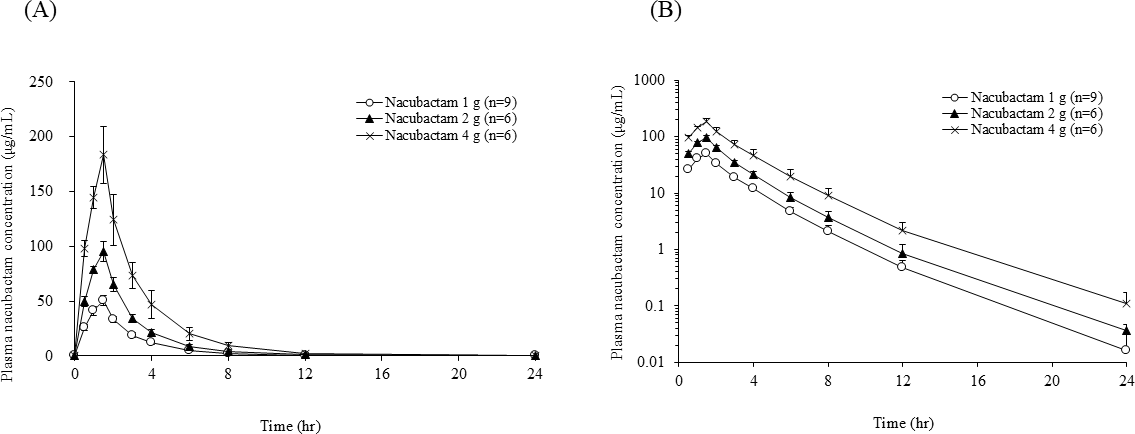
Linear (**A**) and semi-logarithmic (**B**) mean plasma concentration-time profiles of nacubactam after a single infusion of 1, 2, or 4 g over 90 min by dose level (OP0595-2 study).

At the single-dose stage of the OP0595-2 study, plasma concentrations of nacubactam (1, 2, and 4 g doses) peaked at the end of infusion on Day 1 and then rapidly declined (Fig. 3), with similar profiles regardless of dose. The mean elimination half-life (*t*_1/2_) on Day 1 was ranged narrowly from 2.137 to 2.510 h (Tables 3 to 5). Mean total clearance (CL), volume of distribution at steady state (*V*_ss_), and renal clearance (CL_r_) were also constant across dose groups.

**TABLE 3:** Summary statistics of nacubactam pharmacokinetic parameters after intravenous infusion (over 90 min) in the OP0595-2 study^*a*^

Drug	Day	*n*	*C*_max_ (µg/mL)	*t*_max_ (h)	AUC_0–8h_(µg·h/mL)	AUC_0–∞_(µg·h/mL)	*t*_1/2_ (h)	CL (L/h)	Vd_ss_ (L)	CL_r_ (L/h)	Fe_0–24h_ (%)
Nacubactam 1 g	1	9	50.6 ± 4.64	1.500 ± 0.000	129.164 ± 10.230	135.279 ± 11.420	2.137 ± 0.421	7.437 ± 0.601	15.441 ± 1.731	6.628 ± 0.766	88.78 ± 6.33
	9	6	51.3 ± 3.57	1.500 ± 0.000	137.191 ± 8.777	145.311 ± 10.072	2.842 ± 0.153	6.910 ± 0.484	15.309 ± 1.049	6.665 ± 0.543	96.52 ± 7.89
Nacubactam 2 g	1	6	95.1 ± 9.03	1.500 ± 0.000	242.228 ± 20.961	253.217 ± 23.779	2.421 ± 0.090	7.955 ± 0.720	16.118 ± 1.048	5.986 ± 0.475	75.70 ± 8.43
	9	6	99.5 ± 13.5	1.500 ± 0.000	250.533 ± 25.660	263.677 ± 30.055	2.825 ± 0.134	7.664 ± 0.831	15.969 ± 1.837	5.887 ± 0.597	77.62 ± 12.31
Nacubactam 4 g	1	6	184 ± 25.9	1.500 ± 0.000	488.960 ± 76.560	517.000 ± 87.260	2.510 ± 0.083	7.890 ± 1.094	17.465 ± 1.483	6.509 ± 0.864	82.65 ± 5.63

^*a*^ Data are presented as mean ± standard deviation.

**TABLE 4:** Summary statistics of M1 pharmacokinetic parameters after intravenous infusion (over 90 min) of nacubactam in the OP0595-2 study

Drug	Day	*n*	*C*_max_ (µg/mL)	*t*_max_ (h)	AUC_0–8h_ (µg·h/mL)	AUC_0–∞_ (µg·h/mL)	*t*_1/2_ (h)	CL_r_ (L/h)	Fe_0–24h_ (%)
Nacubactam 1 g	1	9	0.562 ± 0.0967	1.667 ± 0.250	2.628 ± 0.386	3.088 ± 0.514	2.392 ± 0.250	8.508 ± 2.781	2.66 ± 0.69
	9	6	0.657 ± 0.0924	1.583 ± 0.204	3.194 ± 0.431	3.806 ± 0.572	2.626 ± 0.230	8.342 ± 1.104	3.24 ± 0.48
Nacubactam 2 g	1	6	1.22 ± 0.119	1.750 ± 0.274	5.644 ± 0.766	6.543 ± 1.108	2.265 ± 0.220	6.825 ± 1.127	2.32 ± 0.58
	9	6	1.40 ± 0.149	1.583 ± 0.204	6.606 ± 1.175	7.781 ± 1.910	2.547 ± 0.574	7.529 ± 1.148	3.01 ± 0.46
Nacubactam 4 g	1	6	2.53 ± 0.362	1.917 ± 0.204	12.518 ± 2.184	14.952 ± 3.268	2.483 ± 0.363	6.331 ± 1.369	2.38 ± 0.23

**TABLE 5:** Summary statistics of M2 pharmacokinetic parameters after intravenous infusion (over 90 min) of nacubactam in the OP0595-2 study^*e*^

Drug	Day	*n*	*C*_max_ (µg/mL)	*t*_max_ (h)	AUC_0–8h_ (µg·h/mL)	AUC_0–∞_ (µg·h/mL)	*t*_1/2_ (h)	CL_r_ (L/h)	Fe_0–24h_(%)
Nacubactam 1 g	1	9	0.0427 ± 0.0101	3.444 ± 0.527	0.175 ± 0.079	NC^*a*^	NC^*a*^	16.022 ± 5.517	0.31 ± 0.10
	9	6	0.0251 ± 0.00165^*c*^	2.600 ± 0.548^*c*^	0.060 ± 0.009^*c*^	NC^*a*^	NC^*a*^	25.979 ±9.369^*c*^	0.17 ± 0.06
Nacubactam 2 g	1	6	0.0769 ± 0.0160	3.833 ± 1.169	0.388 ± 0.095	0.585 ± 0.181^*b*^	3.066 ± 0.069^*b*^	11.508 ± 1.268	0.27 ± 0.05
	9	6	0.0331 ± 0.00568	2.833 ± 0.408	0.108 ± 0.047	0.299^*d*^	3.548^*d*^	22.443 ± 6.105	0.14 ± 0.03
Nacubactam 4 g	1	6	0.132 ± 0.0314	3.333 ± 0.516	0.680 ± 0.168	0.882 ± 0.242^*c*^	3.097 ± 0.315^*c*^	9.894 ± 2.040	0.21 ± 0.03

^*a*^ *n* = 0; NC, not calculated.

^*b*^ *n* = 2.

^*c*^ *n* = 5.

^*d*^ *n* = 1.

^*e*^ *C*_max_, maximum plasma concentration; *t*_max_, time to reach maximum plasma concentration; AUC_0–8h_, area under the plasma concentration-time curve from time zero to 8 h; AUC_0–∞_, area under the plasma concentration-time curve from time zero to infinity; *t*_1/2_, elimination half-life; CL, total clearance; Vd_ss_, volume of distribution at steady state; CL_r_, renal clearance; Fe_0–24h_, fraction (cumulative percentage) of dose excreted in urine within 24 h post-dose.

Following single infusion, nacubactam exposure increased dose-dependently on Day 1. The mean (standard deviation [SD]) maximum plasma concentration (*C*_max_) was 50.6 (4.64), 95.1 (9.03), and 184 (25.9) µg/mL for the 1, 2, and 4 g doses, respectively. Corresponding mean (SD) area under the plasma concentration-time curve from time zero to 8 h (AUC_0–8h_) were 129.164 (10.230), 242.228 (20.961), and 488.960 (76.560) µg∙h/mL; Mean (SD) AUC from time zero to infinity (AUC_0–∞_) was 135.279 (11.420), 253.217 (23.779), and 517.000 (87.260) µg∙h/mL. Though not shown in Tables 3 to 5, power model analysis yielded slopes (95% confidence interval [CI]) for *C*_max_, AUC_0–8h_, and AUC_0–∞_were 0.93 (0.84 to 1.01), 0.95 (0.87 to 1.03), and 0.96 (0.87 to 1.04), respectively.

**Fig 4:**
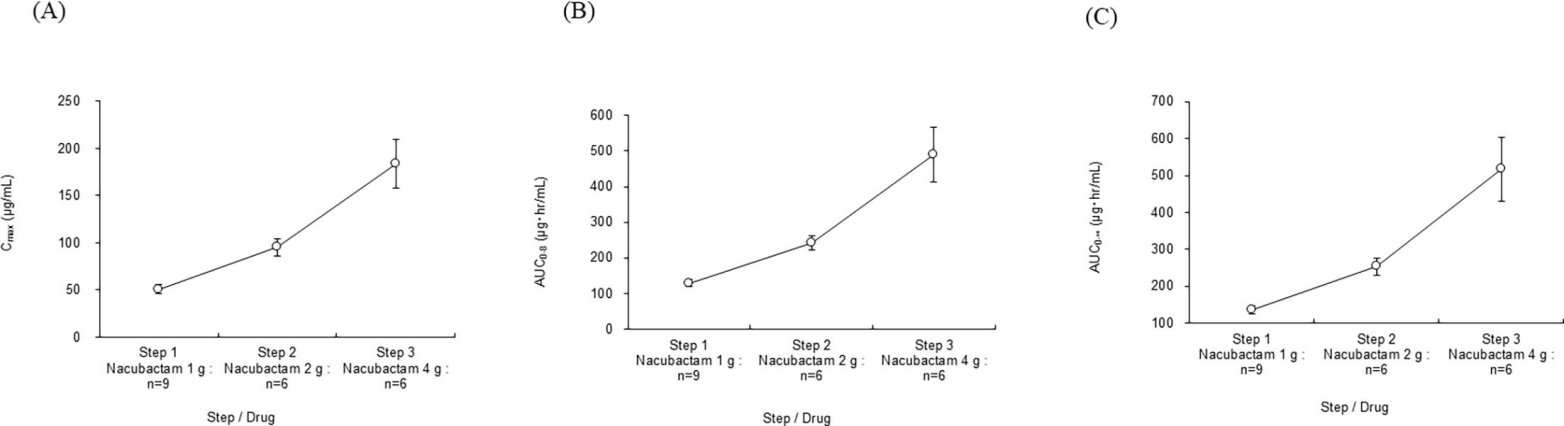
Nacubactam exposure ([**A**] *C*_max_, [**B**] AUC_0–8h_, and [**C**] AUC_0–∞_) after 1, 2, or 4 g nacubactam infusion over 90 min on Day 9 (OP0595-2 study).

At the multiple-dose stage, nacubactam exposure (*C*_max_, AUC_0–8h_, and AUC_0–∞_) on Day 9 increased with dose (Fig. 4) and remained similar to Day 1 for each dose level (Tables 3 to 5). Mean trough plasma concentration (*C*_trough_) from Day 4 to Day 9 was stable, ranging 3.03 to 3.30 μg/mL (1 g) and 5.16 to 5.41 μg/mL (2 g) groups (Table S1).

**Fig 5:**
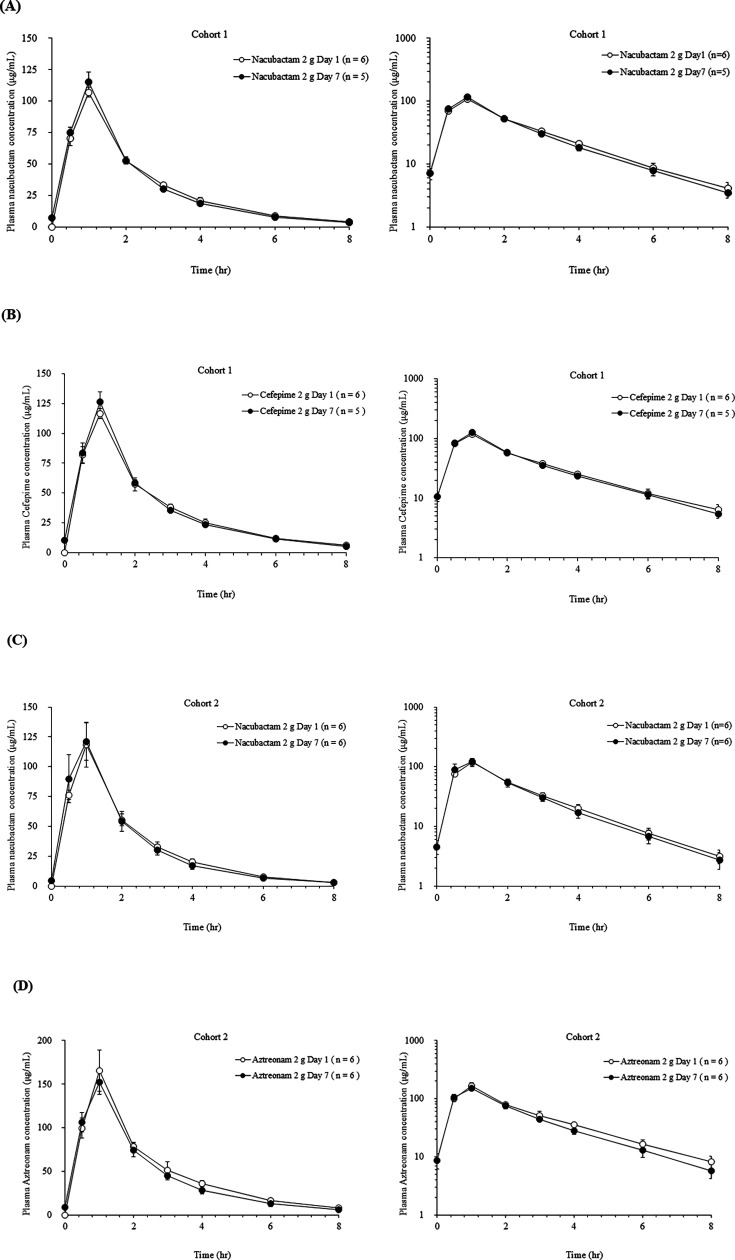
Linear and semi-logarithmic mean plasma concentration-time profiles of nacubactam (2 g per dose) and coadministered β-lactam antibiotics (2 g per dose for each drug) after a single infusion over 60 min on Day 1 and multiple infusions on Day 7 (OP0595-4 study). (**A**) Profiles of nacubactam on Days 1 and 7 in Cohort 1. (**B**) Profiles of cefepime on Days 1 and 7 in Cohort 1. (**C**) Profiles of nacubactam on Days 1 and 7 in Cohort 2. (**D**) Profiles of aztreonam on Days 1 and 7 in Cohort 2.

In the OP0595-4 study, mean plasma concentration-time profiles for nacubactam, cefepime, or aztreonam on Day 7 similar to those on Day 1 (Fig. 5). Nacubactam profiles were also comparable between Cohort 1 and 2. PK parameters are summarized in Table 6. Mean *C*_max_, AUC_0–8h_, and AUC_0–∞_ on Days 1 and 7 were comparable between Cohorts 1 and 2 and aligned closely with observed on Days 1 and 9 in the 2 g group in the OP0595-2 study.

**TABLE 6:** Summary statistics of pharmacokinetic parameters after intravenous infusion (over 60 min) of nacubactam and concomitant drug (2 g per dose for each drug) three times daily for 7 days in the OP0595-4 study^*b*^

Cohort	Measurement	Day	*n*	*C*_max_ (µg/mL)	*t*_max_ (h)	AUC_0-8h_ (µg·h/mL)	AUC_0–∞_ (µg·h/mL)	*t*_1/2_ (h)	CL (L/h)	*V*_ss_ (L)	CL_r_ (L/h)	Fe_0–8h_ (%)^*a*^
1	Nacubactam	1	6	107.0 ± 3.847	1.000 ± 0.000	253.872 ± 8.388	263.841 ± 10.698	1.669 ± 0.135	7.885 ± 0.259	15.994 ± 1.328	NA	79.31 ± 15.10
		7	5	115.4 ± 7.503	1.000 ± 0.000	254.976 ± 13.802	269.910 ± 15.687	2.788 ± 0.162	7.862 ± 0.416	16.110 ± 1.140	4.131 ± 2.907	52.13 ± 36.62
	Cefepime	1	6	116.5 ± 4.231	1.000 ± 0.000	291.382 ± 20.108	309.582 ± 24.966	1.941 ± 0.206	6.891 ± 0.469	15.983 ± 1.077	NA	78.89 ± 16.40
		7	5	126.4 ± 8.204	1.000 ± 0.000	296.153 ± 11.457	321.202 ± 14.116	2.859 ± 0.166	6.761 ± 0.250	16.209 ± 1.411	3.401 ± 2.075	49.66 ± 30.80
2	Nacubactam	1	6	118.4 ± 18.87	1.000 ± 0.000	263.389 ± 25.912	270.163 ± 26.839	1.459 ± 0.103	7.654 ± 0.741	13.800 ± 1.700	NA	85.61 ± 6.29
		7	6	121.0 ± 15.85	1.000 ± 0.000	263.212 ± 38.552	274.227 ± 40.971	2.819 ± 0.133	7.729 ± 1.074	13.892 ± 2.205	6.825 ± 1.986	87.19 ± 18.05
	Aztreonam	1	6	165.5 ± 23.75	1.000 ± 0.000	398.598 ± 36.415	421.381 ± 40.375	1.870 ± 0.226	5.054 ± 0.479	11.654 ± 1.514	NA	63.82 ± 3.78
		7	6	152.2 ± 14.12	1.000 ± 0.000	363.083 ± 36.970	385.757 ± 42.782	2.324 ± 0.099	5.556 ± 0.568	12.072 ± 1.268	3.458 ± 0.849	62.20 ± 13.22

^*a*^ One participant in Cohort 2 was excluded from the analysis on Day 1 because urine samples were not collected from 0 to 4 h.

^*b*^ Data are presented as mean ± standard deviation. Nacubactam and cefepime were administered in Cohort 1, whereas nacubactam and aztreonam were administered in Cohort 2. One participant in Cohort 1 discontinued the study on Day 2. NA, data are not available; Fe_0-8hr_, fraction (cumulative percentage) of dose excreted in urine within 8 hours post-dose. *C*_max_, maximum plasma concentration; *t*_max_, time to reach maximum plasma concentration; AUC_0–8h_, area under the plasma concentration-time curve from time zero to 8 h; AUC_0–∞_, area under the plasma concentration-time curve from time zero to infinity; *t*_1/2_, elimination half-life; CL, total clearance; Vd_ss_, volume of distribution at steady state; CL_r_, renal clearance; Fe_0–24h_, fraction (cumulative percentage) of dose excreted in urine within 24 h post-dose.

#### Metabolites of nacubactam in plasma concentrations (OP0595-2 study)

After single doses of nacubactam (1, 2, and 4 g) on Day 1, mean (SD) *C*_max_ of M1 (open ring analog) was 0.562 (0.0967), 1.22 (0.119), and 2.53 (0.362) µg/mL, respectively. Mean (SD) *C*_max_ of M2 (deaminated ethoxy analog) was 0.0427 (0.0101), 0.0769 (0.0160), and 0.132 (0.0314) µg/mL, respectively. Mean (SD) AUC_0–∞_ of M1 was 3.088 (0.514), 6.543 (1.108), and 14.952 (3.268) µg∙h/mL, respectively. The mean AUC_0-∞_ of M2 was not calculable for the 1 g dose; its mean (SD) value was 0.585 (0.181) µg∙h/mL for the 2 g dose and 0.882 (0.242) µg∙h/mL for the 4 g dose.

After multiple dosing, Mean (SD) *C*_max_ of M1 on Day 9 was 0.657 (0.0924) µg/mL in the 1 g group and 1.40 (0.149) µg/mL in the 2 g group. Mean (SD) *C*_max_ of M2 was 0.0251 (0.00165) and 0.0331 (0.00568) µg/mL, respectively. Mean (SD) AUC_0–∞_ of M1 was 3.806 (0.572) and 7.781 (1.910) µg∙h/mL, respectively. AUC_0–∞_ of M2 could not be calculated in the 1 g group, whereas that was 0.299 µg∙h/mL (*n* = 1) in the 2 g group.

#### Urinary excretion

At the single-dose stage of the OP0595-2 study, mean fraction excreted in urine within 24 h post-dose (Fe_0–24h_) of nacubactam on Day 1 was 88.78%, 75.70%, and 82.65% for 1, 2, and 4 g dose, respectively (Tables 3 to 5). Nacubactam was largely excreted unchanged in urine. Although not shown in Tables 3 to 5, mean Fe_0–24h_ of M1 on Day 1 were 2.66%, 2.32%, and 2.38%, while those of Fe_0-24h_ of M2 was 0.31%, 0.27%, and 0.21% for 1, 2, and 4 g dose, respectively. Overall, 91.75%, 78.30%, and 85.24% of the administered doses (1, 2, and 4 g) were excreted as nacubactam or metabolites within 24 h.

At the multiple-dose stage (Day 9), mean Fe_0–24h_ of nacubactam was 96.52% (1 g group) and 77.62% (2 g group) (Tables 3 to 5). Corresponding mean Fe_0–24h_ of M1 on Day 9 was 3.24% and 3.01%, and for Fe_0–24h_ of M2 was 0.17% and 0.14%. Total urinary excretion of nacubactam and metabolites reached 99.93% and 80.76% for 1 g and 2 g doses, respectively, within 24 h.

In the OP0595-4 study, mean urinary excretion fraction of nacubactam within 8 h post-dose (Fe_0–8h_) on Day 1 were 79.31% (Cohort 1) and 85.61% (Cohort 2). On Day 7, mean Fe_0–8h_ was 52.13% and 87.19%, respectively (Table 6). Metabolite fractions were not assessed in this study. Fraction of dose excreted in urine (Fe) is ~79% for nacubactam and cefepime on day 1 and ~50% on Day 7 in study OP0595-4. Two of the six subjects had low urinary excretion rates for nacubactam and cefepime on Day 7 only.

#### Pharmacokinetics of cefepime and aztreonam (OP0595-4 study)

PK parameters of cefepime and aztreonam are shown in Table 6. Cefepime exposure on Day 7 was comparable to Day 1, with mean (SD) *C*_max_ were 116.5 (4.231) µg/mL on Day 1 and 126.4 (8.204) µg/mL on Day 7. Mean (SD) AUC_0–8h_ was 291.382 (20.108) and 296.153 (11.457) µg·h/mL, respectively. Mean (SD) AUC_0-∞_ was 309.582 (24.966) and 321.202 (14.116) µg·h/mL, respectively. The mean Fe_0–8h_ was 78.89% and 49.66%, respectively.

Similarly, aztreonam exposure on Day 7 was comparable to Day 1, with mean (SD) *C*_max_ was 165.5 (23.75) µg/mL on Day 1 and 152.2 (14.12) µg/mL on Day 7. Mean (SD) AUC_0–8h_ was 398.598 (36.415) and 363.083 (36.970) µg·h/mL, respectively. Mean (SD) AUC_0-∞_ was 421.381 (40.375) and 385.757 (42.782) µg·h/mL, respectively. The mean Fe_0–8h_ was 63.82% and 62.20%, respectively. Accumulation ratio of nacubactam (with cefepime/aztreonam), cefepime, or aztreonam (*C*_max_ and AUC_0–8h_) was 1.05 (108.26/103.02 and 101.10/99.62), 1.06 (109.85 and 103.79), or 1.02 (92.71 and 91.15).

### Safety

#### TEAEs

In the OP0595-2 study, no TEAEs occurred during the single-dose stage. At the multiple-dose stage, one participant in the 1 g group experienced dizziness, and another in the 2 g group reported vessel puncture site erythema and puncture site pain. All events were mild and unrelated to the study drug. No TEAEs were observed in the 4 g or placebo groups (Table 7).

**TABLE 7:** Frequency of TEAEs classified in the OP0595-2 study (multiple dose stage)

	Step 1 Nacubactam 1 g × 3/day			Step 2 Nacubactam 2 g × 3/day		
		*n* = 9			*n* = 6	
	Number of incidence	Number of subjects with incidence	Incidence rate (%)	Number of incidence	Number of subjects with incidence	Incidencerate (%)
General disorders and administration site conditions	0	0	(0.0)	2	1	(16.7)
Puncture site pain	0	0	(0.0)	1	1	(16.7)
Vessel puncture site erythema	0	0	(0.0)	1	1	(16.7)
Nervous system disorders	1	1	(11.1)	0	0	(0.0)
Dizziness	1	1	(11.1)	0	0	(0.0)
					MedDRA/J Ver.19.1	

In the OP0595-4 study, three participants receiving nacubactam with cefepime experienced mild TEAEs: increased transaminases, hematuria, and seborrheic dermatitis, each in one participant. These events were considered unrelated to nacubactam or cefepime. Notably, seborrheic dermatitis appeared on Day 1, causing study discontinuation on Day 2; it resolved by Day 8 with hydrocortisone ointment treatment. No TEAEs were reported in the nacubactam with aztreonam or placebo group (Table 8).

**TABLE 8:** Frequency of TEAEs classified in the OP0595-4 study

Cohort	Drug	*n*	Adverse events	*N* events	*N* (%) subjects	Incidencerate (%)
			SOC (system organ class)			
			PT (preferred term)			
1	OP0595 : 2 g	6	Investigations	1	1	(16.7)
	With Cefepime : 2 g		Transaminases increased	1	1	(16.7)
			Renal and urinary disorders	1	1	(16.7)
			Hematuria	1	1	(16.7)
			Skin and subcutaneous tissue disorders	1	1	(16.7)
			Seborrheic dermatitis	1	1	(16.7)
					MedDRA/J Ver.23.0	

#### Laboratory tests, weight, vital signs, electrocardiogram assessments, and electrocardiogram assessments

In both studies, no clinically meaningful changes were observed in the laboratory test results, weight, vital signs (including body temperature, blood pressure, and pulse rate), or electrocardiogram (ECG) assessments.

## DISCUSSION

At the single-dose stage of the OP0595-2 study, nacubactam’s mean *C*_max_, AUC_0–8h_, and AUC_0–∞_ increased dose-dependently following infusion 1, 2, and 4 g, with a consistent mean half-life (*t*_1/2_) of approximately 2 h across dose. Power model analysis showed that the 95% *CIs* of the slopes for *C*_max_, AUC_0–8h_, and AUC_0–∞_ included 1, indicating linear increases in systemic exposure. During the multiple-dose stage, mean trough concentrations (*C*_trough_) stabilized after Day 4, with mean *C*_max_, AUC_0–8h_, and AUC_0–∞_ remaining similar between Days 1 and 9. These findings suggest rapid attainment of steady state with minimal accumulation. After IV administration of nacubactam, metabolites were found in plasma and urine. The metabolites observed were M1 (open-ring form) which has no inhibitory activity, and M2 (deaminoethoxy form) has inhibitory activity (12). Metabolites (M1 and M2) were detected at low levels relative to parent drug, and urinary excretion analyses indicated that nacubactam was primarily eliminated unchanged via the kidneys with minimal metabolism. Although M2 has inhibitory activity, M2 was present at exposures of <0.5% of corresponding nacubactam exposures.

In the OP0595-4 study, nacubactam was coadministered with cefepime or aztreonam. Since no monotherapy arms were included, potential drug-drug interaction could not be fully assessed. However, pharmacokinetic parameters (*C*_max_, AUC_0–8h_, and AUC_0–∞_) of nacubactam on Days 1 and 7 were similar between cohorts despite different concomitant drugs to those seen in the OP0595-2 study. The pharmacokinetic parameters of cefepime and aztreonam were not different from those previously reported (15, 16). There were no prespecified equivalence/no-effect bounds for the DDI assessment when nacubactam is co-administered with cefepime/aztreonam. However, there is thought to be no drug-drug interaction in terms of pharmacokinetics or safety, and no signs of interaction were observed in this study.

These results align with prior phase 1 studies in Western populations (12), which demonstrated linear pharmacokinetics of nacubactam over a wide dose range (0.05 to 8 g single dose; 1 to 4 g multiple doses) with a short *t*_1/2_ around 2 h. Those studies also showed minimal metabolites formation and predominant renal excretion of unchanged drug. Additionally, coadministration of nacubactam with meropenem (2 g per dose for each drug) for 6 days did not alter the pharmacokinetics of either drug.

Although the exclusion criteria, such as the subjects’ BMI, differed between the two studies, this was not thought to affect the evaluation. Also, although the safety MedDRA versions were different, this was not thought to affect the evaluation.

Regarding safety, nacubactam alone or combined with cefepime or aztreonam was generally well tolerated. In the OP0595-2 study, three participants experienced mild TEAEs occurred in participants receiving nacubactam with cefepime, also considered unrelated to treatment; no TEAEs were reported in the aztreonam or placebo groups. No clinically meaningful changes were observed in the laboratory test, vital signs, or ECG assessments in either study.

These findings are consistent with earlier phase 1 studies (12) where nacubactam alone or with meropenem was well tolerated, with no serious TEAEs or dose-related safety concerns.

In efficacy, combining nacubactam with cefepime or nacubactam with aztreonam is thought to enable the treatment of all Ambler class CRE (including some Pseudomonas aeruginosa strains). The results of Phase III trials demonstrate the effectiveness of these combination treatments. These studies confirmed that nacubactam has a favorable PK profile to support efficacy assessment when used in combination with β-lactams. In addition, no ethnic differences in PK were observed between Westerners and Japanese, suggesting that dose adjustment is unnecessary.

This study (OP0595-4) was discontinued due to the COVID-19. This did not affect the completeness of multiple-dose PK assessment and steady-state evaluations compromised.

This study was limited to healthy adult males and did not evaluate the effects of patient populations, women, elderly people, with kidney or liver impairment, and those with comorbidities external or internal factors related to disease-related physiological changes. Due to the small sample size, single-center, and short-term study, the power to detect rare adverse events and the evaluation of long-term safety were insufficient. Furthermore, PK and safety data obtained under controlled conditions (e.g., fasting, standardized meal) may not be generalizable to the diverse lifestyles and concomitant medication environments experienced in clinical practice. Therefore, while these results provide an initial basis for dose setting in Japan, they require further confirmation in infected patient populations.

In conclusion, nacubactam, whether administered alone or combined with cefepime or aztreonam, demonstrated linear pharmacokinetics with rapid steady-state attainment and minimal accumulation in healthy Japanese participants, primarily eliminated unchanged in urine and well tolerated. Safety profiles were favorable and comparable to previous studies in Western populations, suggesting no ethnic differences. These results support further clinical development of nacubactam,

## MATERIALS AND METHODS

### Study oversight

OP0595-2 study was conducted at Showa University Karasuyama Hospital from 17 January to 20 July 2017, while OP0595-4 study was conducted at SOUSEIKAI Sumida Hospital between 6 March and 17 April 2020. Both studies were performed in accordance with the Declaration of Helsinki and Good Clinical Practice guidelines. Ethical approval was obtained from the institutional review board of respective sites. All participants provided written informed consent prior to enrollment.

### Study population

Japanese healthy male volunteers aged between 20 and 39 years with a body mass index (BMI) within 17.6 and 24.6 kg/m^2^ for the OP0595-2 study, and ≥18.5 and <25.0 kg/m^2^ for the OP0595-4 study, were eligible if they had no clinically significant abnormalities. Key exclusion criteria included use of any medication (except for topical agents without systemic effects) within 7 days before dosing, or a history or current diagnosis of allergic symptoms, or cardiac, hepatic, renal, gastrointestinal, respiratory, vascular, or hematological functions. Participants with a QTc interval of 450 msec or longer, calculated by Fridericia’s formula on 12-lead ECG, were also excluded.

The reason for including only healthy adult males was to eliminate the influence of factors such as gender and body size when confirming the pharmacokinetics, safety, and tolerability in the Japanese population based on overseas data.

### Study treatment

OP0595-2 study was conducted in three steps. In Steps 1 and 2, participants were hospitalized to the study site from 2 days before dosing (Day −2) until Day 10. They received a single dose of the study drug on Day 1, followed by administration three times daily every 8 h from Day 3 to Day 8, with a final single dose on Day 9. Assessments continued through Day 10, and participants returned for a follow-up visit on Day 16. In Step 3, participants were hospitalized from Day 1 until Day 3, receiving a single dose on Day 1 and returning for follow-up on Day 8. Dosing on Day 2 was skipped to evaluate the pharmacokinetics and safety of a single dose on Day 1. In addition, the single dose on Day 9 was set to evaluate pharmacokinetics until elimination.

In all steps, participants were randomized in a 3:1 ratio to receive nacubactam or placebo at a dose of 1 g (Step 1), 2 g (Step 2), and 4 g (Step 3). Both nacubactam and placebo were administered by intravenous infusion over 90 min. Each step planned to enroll eight participants (six receiving nacubactam and two receiving placebo).

The OP0595-4 study included two cohorts, which participants hospitalized from Day −1 to Day 8, and a follow-up visit on Day 14. Participants were randomized in a 3:1 ratio to receive nacubactam (2 g per dose) with the concomitant drug (2 g per dose) or placebo. The concomitant drug was cefepime in Cohort 1 and aztreonam in Cohort 2. All drugs and placebo were administered intravenously over 60 min, three times daily every 8 h for 7 days, with the final dose given on the morning of Day 7. Each cohort was planned to enroll eight participants (six receiving nacubactam with concomitant, and two receiving placebo).

In study OP0595-2, the infusion duration was initially set at 90 min. Subsequently, nonclinical studies were conducted to determine the optimal infusion time, and a 60-min infusion duration was selected for Study OP0595-4. The 2 g dose was selected to provide a sufficient dose to inhibit the concomitant beta-lactam drug and to confirm the tolerability of nacubactam.

### PK measurements

In Steps 1 and 2 of OP0595-2 study, blood samples for PK analysis were collected at the following time points: pre-dose and 0.5, 1, 1.5, 2, 3, 4, 6, 8, 12, and 24 h after start of the infusion on Day 1; immediately before each morning infusion from Day 3 to Day 8; and on Day 9, immediately before at and at 0.5, 1, 1.5, 2, 3, 4, 6, 8, 12, and 24 h after infusion. In Step 3, samples were collected at the same points following the single dose on Day 1. Plasma concentrations of nacubactam and its metabolites (M1 and M2) were measured.

For the OP0595-4 study, blood samples for PK analysis were obtained at pre-dose and at 0.5, 1, 2, 3, 4, 6, and 8 h after infusion on Day 1; at pre-dose on Day 4; and on Day 7, at pre-dose and at 0.5, 1, 2, 3, 4, 6, 8, 12, and 24 h post infusion. In this study, the plasma concentrations of nacubactam and concomitant drugs were measured.

Urine samples for PK analysis were also collected in both studies. In the OP0595-2 study, collection occurred at 0–4, 4–8, 8–12, and 12–24 h after infusion on Days 1 and 9 of Steps 1 and 2, and on Day 1 of Step 3. The cumulative percentages of nacubactam and its metabolites excreted in urine were calculated. In the OP0595-4 study, urine samples were collected at 0–4 and 4–8 h after infusion on Day 1, and 0–4, 4–8, 8–12, and 12–24 h after infusion on Day 7, to calculate the cumulative urinary excretion of nacubactam and concomitant drugs.

In both studies, liquid chromatography tandem mass spectrometry was used to measure concentrations of nacubactam, its metabolites, and any concomitant drugs in plasma and urine.

In the OP0595-2 study, plasma and urine concentrations of nacubactam and its metabolites (M1 and M2) were analyzed by LC-MS/MS. Plasma concentrations were analyzed using a calibration curve in the range of 0.02–5 µg/mL, and urinary concentrations using a calibration curve in the range of 0.01–10 µg/mL.

In the OP0595-4 study, plasma and urine concentrations of nacubactam, cefepime, and aztreonam were measured using LC-MS/MS. The calibration curve ranges were 0.02–5 µg/mL, 0.01–10 µg/mL, and 0.01–10 µg/mL for plasma concentrations and 0.04–40 µg/mL, 0.1–50 µg/mL, and 0.1–50 µg/mL for urinary concentrations, respectively.

In both studies, the bioanalysis met the standards of the ICH M10 guideline. The accuracy of the QC samples was within an acceptable range as shown in the table.

### Safety assessments

Safety assessments included monitoring for TEAEs, laboratory tests (hematology, serum chemistry, and urinalysis), and physiological tests (height, weight, body mass index, body temperature, blood pressure, and pulse rate, and resting 12-lead ECG recording). TEAEs were defined as any unexpected signs, symptoms, or illness that developed in participants who received the study drug or concomitant drug. Cording of TEAEs was based on the Medical Dictionary for Regulatory Activities Japanese: version 19.1 for the OP0595-2 study and version 23.0 for the OP0595-4 study.

### Statistical analysis

In both studies, the safety analysis set included all participants who received at least one dose of the study drug. The PK analysis set comprised all participants who received nacubactam (with or without the concomitant drug) and had valid PK measurements. Baseline characteristics and TEAEs were summarized descriptively.

PK parameters were calculated using non-compartmental analysis using Phoenix WinNonlin (Certara USA Inc., Princeton, NJ) version 6.3 for the OP0595-2 study and version 8.1 for the OP0595-4 study. All data analyses were conducted with SAS version 9.4 (SAS Institute, Cary, NC).

For the OP0595-2 study, a power model was used to evaluate dose proportionality for nacubactam exposure (*C*_max_, AUC_0–8h_, and AUC_0–∞_). This model estimated the slope and its 95% confidence interval (CI) using the following equation: ln (*C*_max_, AUC_0–8h_, or AUC_0–∞_) = *α* + *β* ln (dose), where *α* was the intercept and *β* was the slope.

## Data Availability

The data sets generated during and/or analyzed during the current study are available from the corresponding author upon reasonable request.

## SUPPLEMENTAL MATERIAL

Table S1 aac.01770-25-s0001.docx Summary of plasma trough level of OP0595 in the OP0595-2 study.

ASM does not own the copyrights to Supplemental Material that may be linked to, or accessed through, an article. The authors have granted ASM a non-exclusive, world-wide license to publish the Supplemental Material files. Please contact the corresponding author directly for reuse.
