# Supplementary material for: Phase I studies assessing safety and pharmacokinetics of nacubactam administered alone or in combination with cefepime or aztreonam in Japanese healthy participants
Source: Antimicrob Agents Chemother. 2026 Apr 13;70(5):e01770-25. doi: 10.1128/aac.01770-25 (PMC13148055; doi:10.1128/aac.01770-25)
Supplement: Table S1 — Summary of plasma trough level of OP0595 in the OP0595-2 study. [file aac.01770-25-s0001.docx]

# Supplement

**Table S1** Summary of plasma trough level of OP0595 in the OP0595-2 study

| Step/Study drug | Descriptive statistics |  | | | | | |
| --- | --- | --- | --- | --- | --- | --- | --- |
|  |  | Day 4 | Day 5 | Day 6 | Day7 | Day8 | Day9 |
| Step 1 | n | 6 | 6 | 6 | 6 | 6 | 6 |
| Nacubactam 1 g ×3/日 | Mean | 3.22 | 3.13 | 3.30 | 3.03 | 3.08 | 3.15 |
|  | SD | 0.443 | 0.438 | 0.420 | 0.317 | 0.473 | 0.528 |
| Step 2 | n | 6 | 6 | 6 | 6 | 6 | 6 |
| Nacubactam 2 g ×3/日 | Mean | 5.16 | 5.27 | 5.38 | 5.36 | 5.41 | 5.32 |
|  | SD | 0.777 | 1.42 | 1.45 | 1.16 | 1.14 | 1.53 |

NC：Not calculated
